# MRI-based proton density fat fraction combined with adipose tissue for identifying metabolic dysfunction-associated steatohepatitis

**DOI:** 10.1186/s13244-026-02444-9

**Published:** 2026-10-29

**Authors:** Yiming Yang, Simei Lai, Xiaohuan Li, Dongjing Zhou, Zhiyuan Chen, Zelan Ma, Xiaoting Zhang, Yupin Liu

**Affiliations:** 1 Department of Radiology, The Second Affiliated Hospital of Guangzhou University of Chinese Medicine, Guangzhou, 510120 Guangdong, China; 2 The Second Clinical College of Guangzhou University of Chinese Medicine, Guangzhou, 510120 Guangdong, China

**Keywords:** Magnetic resonance imaging, Proton density fat fraction, Adipose tissue, Metabolic dysfunction-associated steatohepatitis

## Abstract

**Objectives:**

To develop a non-invasive diagnostic model combining magnetic resonance imaging (MRI)-derived proton density fat fraction (PDFF) and adipose tissue for identifying metabolic dysfunction-associated steatohepatitis (MASH).

**Materials and methods:**

A total of 197 consecutive patients with metabolic dysfunction-associated steatotic liver disease (MASLD) who underwent liver biopsy, MRI-PDFF, and transient elastography (TE) were retrospectively enrolled. Independent predictors of MASH were identified using univariate and multivariate logistic regression analyses. A combined model was constructed and internally validated via 1000 bootstrap resamples. The diagnostic performance was compared with that of established non-invasive tests (NITs).

**Results:**

Multivariable logistic regression analyses demonstrated that the PDFF and total adipose tissue index (TATI) were independent risk factors for MASH. The MRI-based PDFF-adipose tissue (MR-PAT) model achieved an area under the receiver operating characteristic curve (AUC) of 0.866 for MASH identification. Bootstrap internal validation yielded an AUC of 0.870. The performance of the models was consistent across clinically relevant subgroups. The diagnostic performance of the MR-PAT model for MASH identification was significantly better than that of all the established NITs (all *p* < 0.05).

**Conclusion:**

The MR-PAT model, combining MRI-derived PDFF and TATI, provides an accurate and robust non-invasive tool for identifying MASH. It outperforms widely recognized NITs and holds promise for risk stratification in patients with MASLD.

**Key Points:**

***Question*** MASH is a critical histological endpoint of MASLD, but reliable non-invasive tools for its assessment remain inadequate.

***Findings*** The MR-PAT model integrating MRI-based PDFF and adipose tissue quantification demonstrates robust and excellent diagnostic performance for identifying MASH.

***Critical Relevance Statement*** The MR-PAT model leverages hepatic steatosis and the adipose tissue burden to elucidate the pathophysiological mechanisms driving steatohepatitis. It can serve as an accurate, non-invasive tool for identifying MASH and holds promise for risk stratification in patients with MASLD.

**Graphical Abstract:**

**Figure:**
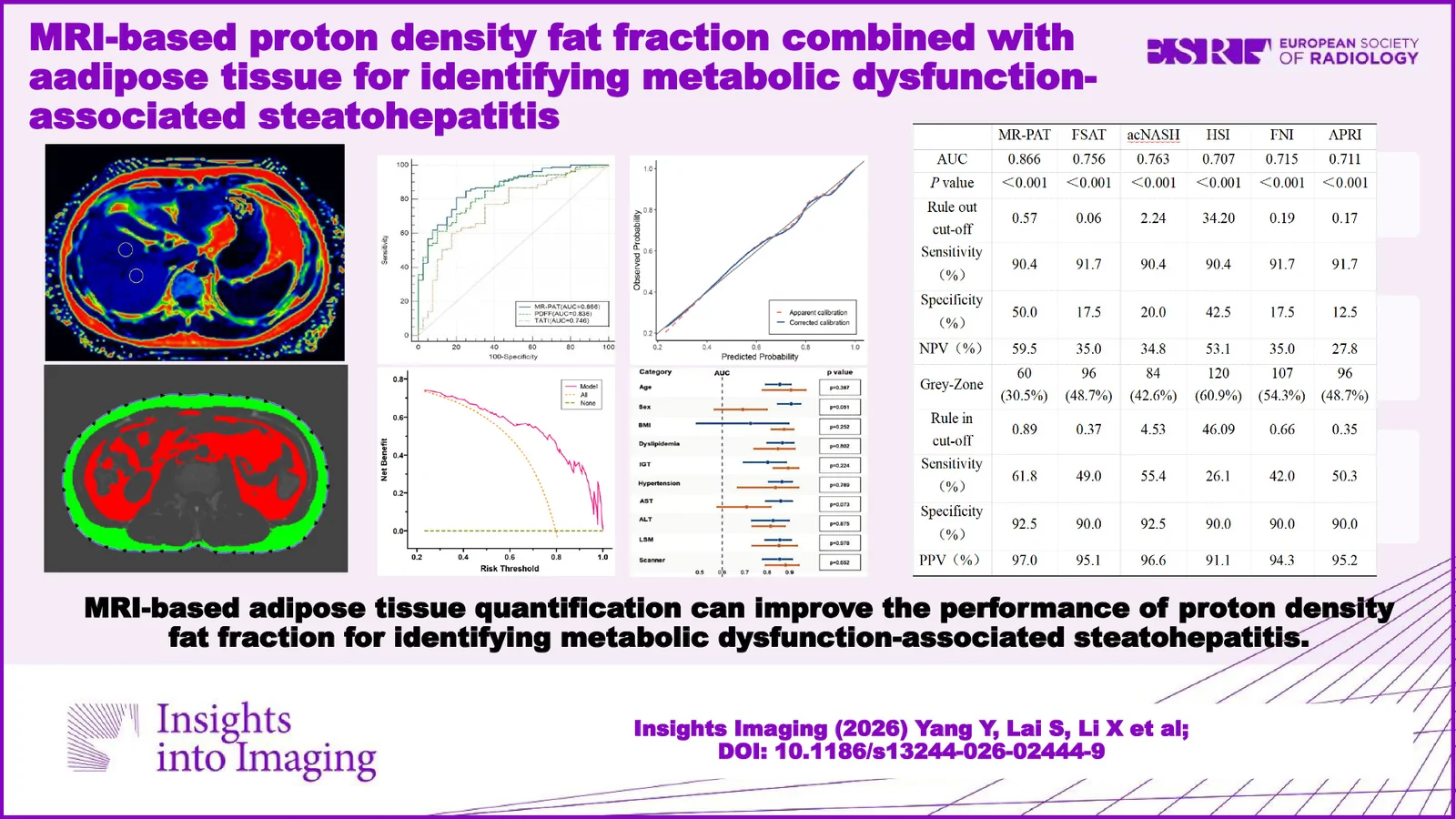

## Introduction

Metabolically dysfunction-associated steatotic liver disease (MASLD) is the leading cause of chronic liver disease worldwide, with recent studies indicating that it may affect more than 38% of adults and 7%–14% of children globally [1]. MASLD encompasses a continuous disease spectrum, ranging from metabolic dysfunction‑associated steatotic liver (MASL) to metabolic dysfunction‑associated steatohepatitis (MASH) and the more advanced stage of at‑risk MASH (*F* ≥ 2) [2, 3]. Individuals who present with MASH, especially at‑risk MASH, exhibit a substantially increased risk of adverse outcomes, such as hepatocellular carcinoma (HCC), hepatic decompensation and liver‑related mortality [4, 5].

Liver biopsy remains the reference standard for risk stratification in MASLD. However, as an invasive approach, liver biopsy has inherent complications and is limited by procedure-related hazards and sampling bias [6]. Recently, imaging biomarkers developed based on transient elastography (TE) techniques, represented by the FibroScan-AST (FAST) score, have been used to identify patients with at‑risk MASH and select candidates for pharmacotherapy [7]. Nevertheless, emerging evidence from clinical trials indicates that personalized therapeutic strategies yield heterogeneous and often divergent effects on key histological endpoints, including MASH resolution and fibrosis regression [8–10]. These findings underscore the demand for refined risk stratification and tailored treatment approaches in clinical practice, thereby avoiding confusion concerning distinct histological endpoints and disease processes.

Magnetic resonance imaging (MRI)-based proton density fat fraction (PDFF) is widely recognized as a highly precise non-invasive method for assessing the full spectrum of hepatic steatosis [11]. Emerging studies suggest that MRI-PDFF plays a valuable role in the identification of steatohepatitis [12, 13]. However, compared with isolated steatosis, MASH encompasses a more complex pathological entity. Adipose tissue is the primary lipid storage depot and acts as the largest endocrine organ to secrete adipokines, which modulate hepatic metabolism and serve as a key driver in the development of MASLD [14, 15]. While prior studies have linked waist circumference to the presence of MASH, the association between adipose-related body composition and MASH has not been fully elucidated [16].

This study aimed to evaluate the value of MRI-based adipose tissue and to combine it with PDFF in identifying patients with MASH, thereby facilitating more precise risk stratification in MASLD.

## Materials and methods

### Subjects

This retrospective study received approval from the Ethics Committee of the Second Affiliated Hospital of Guangzhou University of Chinese Medicine (BE2023-204-01), and the requirement for written informed consent was waived. Data from patients who underwent MRI-PDFF, TE, and liver biopsy at our institute between October 10, 2017, and October 21, 2024, were retrospectively reviewed. All TEs, MRI examinations, and liver biopsies (including histology performed for the evaluation of hepatic steatosis, lobular inflammation, hepatocellular ballooning, and fibrosis) were performed for clinical purposes unrelated to this investigation. The inclusion criteria were as follows: ① age > 18 years; ② MRI‑PDFF and TE examination performed within one month prior to liver biopsy; ③ presence of at least one of the following cardiometabolic risk factors: (I) body mass index (BMI) ≥ 23 kg/m²; (II) fasting glucose (Glu) ≥ 5.6 mmol/L or hemoglobin A1c (HbA1c) levels ≥ 5.7% or treatment for type 2 diabetes; (III) blood pressure ≥ 130/85 mmHg or receiving antihypertensive medication; and (IV) triglyceride (TG) levels ≥ 150 mg/dL or high-density lipoprotein (HDL) levels < 1.0/1.3 mg/dL or receiving lipid-lowering therapy. The exclusion criteria were as follows: ① complicated liver disease, other than MASLD (*n* = 14); ② excessive alcohol consumption (> 20 g/day for men or > 10 g/day for women) within 1 year prior to recruitment (*n* = 8); ③ use of any medications with hepatotoxic potential (*n* = 3); and ④ previous or current malignancies (*n* = 3).

### MRI examination

All participants underwent chemical shift-encoded MRI (CSE-MRI) using two 3.0-T MRI systems equipped with either an 18-channel phased-array coil (Prisma; Siemens Healthineers, Germany) or an 8-channel Atlas SPEEDER phased-array coil (Titan; Canon Medical Systems, Japan). All the subjects fasted for at least 8 h prior to MRI scanning. The detailed acquisition parameters are listed in Supplementary Methods S1.

### Measurement of hepatic fat fraction

**Fig. 1:**
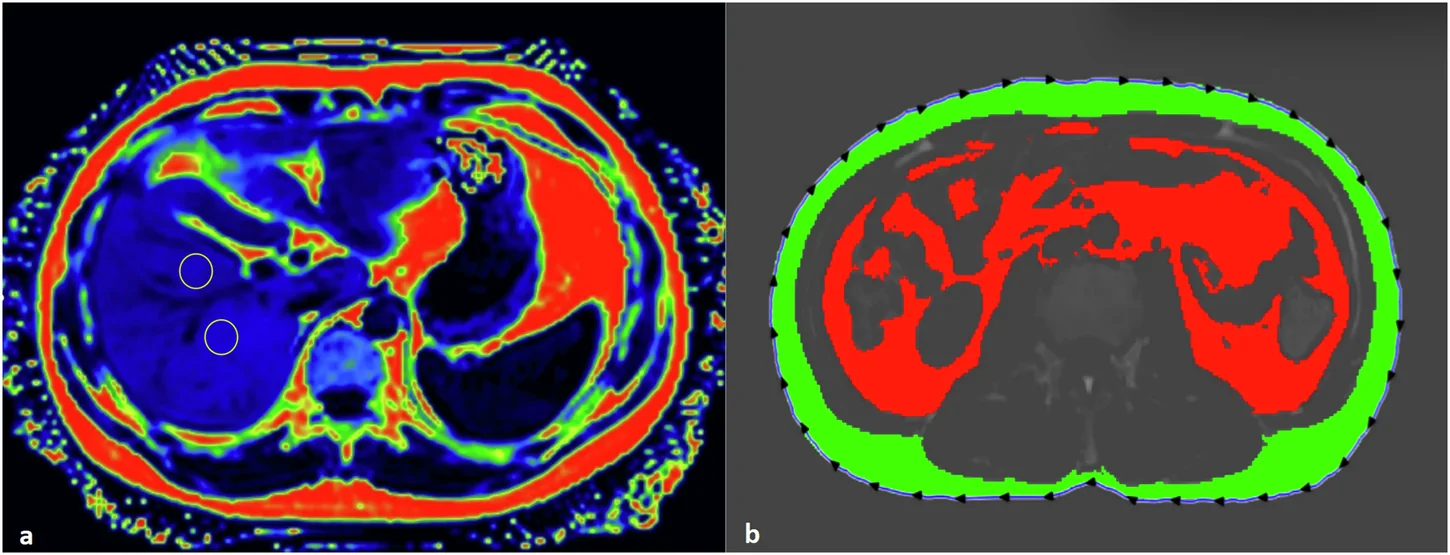
A 56-year-old male with MASLD. **a** Example of ROI delineation for PDFF; **b** measurement diagram of visceral adipose tissue area, subcutaneous adipose tissue, and waist circumference

After image acquisition, in-phase, out-phase, fat-only, and water-only images were reconstructed in sequence, and fat fraction maps were generated automatically. MRI-PDFF values were measured by two experienced radiologists who placed regions of interest (ROIs) on the fat fraction maps and who were blinded to all the clinical and histopathological data. Two circular ROIs of 1.5–2.0 cm^2^ were placed on three consecutive 5 mm thick sections centered on the right branch of the portal vein, with a total of six ROIs obtained. To match the liver biopsy location, the ROIs were placed on the liver parenchyma approximately 2 cm from the liver edge, avoiding bile ducts, large vessels, focal lesions, and artefacts (Fig. 1a). The mean of the six ROI‑derived MRI-PDFF measurements was taken as the patient’s hepatic fat fraction.

### MRI body assessments

MRI body assessments were performed on fat-only images at the third lumbar vertebra (L3) level using SliceOmatic software by a radiologist with 11 years of experience (Fig. 1b). Visceral adipose tissue (VAT) denotes the intra-abdominal fat within the abdomen, whereas subcutaneous adipose tissue (SAT) represents subcutaneous fat external to the abdominal muscles. Adipose tissue areas were semiautomatically segmented via the region-growing mode. The total adipose tissue (TAT) area was calculated as the sum of the VAT area and the SAT area. After normalization by height squared, these adipose tissue areas were presented as the subcutaneous adipose tissue index (SATI), visceral adipose tissue index (VATI), and total adipose tissue index (TATI), respectively. Waist circumference measurements were also obtained using the snake mode.

### TE measurement

TE was performed by a trained hepatologist blinded to clinical and imaging data using a FibroScan (M Probe; Echosens, France). Patients were asked to fast for ≥ 3 h prior to the examination. Liver stiffness measurement‌ (LSM) and controlled attenuation parameter (CAP) results were considered unreliable if the number of successful measurements was < 10, the success rate was < 60%, or the interquartile range/median was ≥ 30%. After ten consecutive successful measurements, the median of the valid values was taken as the liver LSM value and CAP value.

### Laboratory indicators

Clinical data (age, sex, BMI, blood pressure) and laboratory data (TG, total cholesterol (TC), HDL, low-density lipoprotein (LDL), Glu, HbA1c, creatinine (Cr), estimated glomerular filtration rate (eGFR), aspartate aminotransferase (AST), alanine aminotransferase (ALT), total bilirubin (TBIL), albumin (Alb), alkaline phosphatase (ALP), and gamma-glutamyl transferase (GGT)) were obtained from the electronic medical records.

Established non-invasive tests (NITs), including the FAST score, AST-to-creatinine ratio index (acNASH), hepatic steatosis index (HSI), fibrotic NASH index (FNI), and AST-to-platelet ratio index (APRI), were calculated according to previously published formulas [7, 17–19].

### Histological evaluation

Liver biopsy was performed by a hepatologist with 15 years of work experience under ultrasound guidance using a 16-gauge needle. The puncture site was the bare area of the liver, approximately 2 cm away from the liver edge at the level of the main trunk of the right portal vein, and the length of the puncture sample was at least 1.5 cm. The samples were evaluated by a pathologist with 10 years of work experience in accordance with the non-alcoholic fatty liver disease activity score (NAS) system. MASH was defined as a total NAS ≥ 4 points, with each score of steatosis, ballooning, and lobular inflammation ≥ 1 point, with or without fibrosis [20]. MASLD that did not meet the criteria for MASH was defined as metabolic dysfunction–associated steatotic liver (MASL) [21].

### Statistical analyses

Continuous variables are expressed as the mean ± standard deviation for normally distributed data or the median with interquartile range for nonnormally distributed data. Comparisons of normally distributed continuous variables between the MASH and MASL groups were performed using the independent samples *t*-test, while the Mann–Whitney *U*-test was applied for nonnormally distributed variables. Categorical variables are presented as frequencies (percentages) and were compared using the chi-square test. Univariable and multivariable logistic regression analyses were employed to construct a diagnostic model for MASH. The model was internally validated using 1000 bootstrap samples. The area under the receiver operating characteristic curve (AUROC) was used to assess the discriminatory performance of the combined model for identifying MASH. Calibration curves were employed to evaluate model calibration, and decision curve analysis (DCA) was applied to estimate the clinical usefulness of the model. Sensitivity analyses were conducted stratified by sex, age, BMI, atherogenic dyslipidemia, impaired glucose tolerance (IGT), ALT, AST, LSM, and scanner. The DeLong test was adopted to compare the AUCs between the proposed model, PDFF, and NITs. All the statistical analyses were performed using SPSS version 20.0.0 or R version 4.0.5. *p* < 0.05 was considered to indicate statistical significance.

## Results

### Patient characteristics

**Fig. 2:**
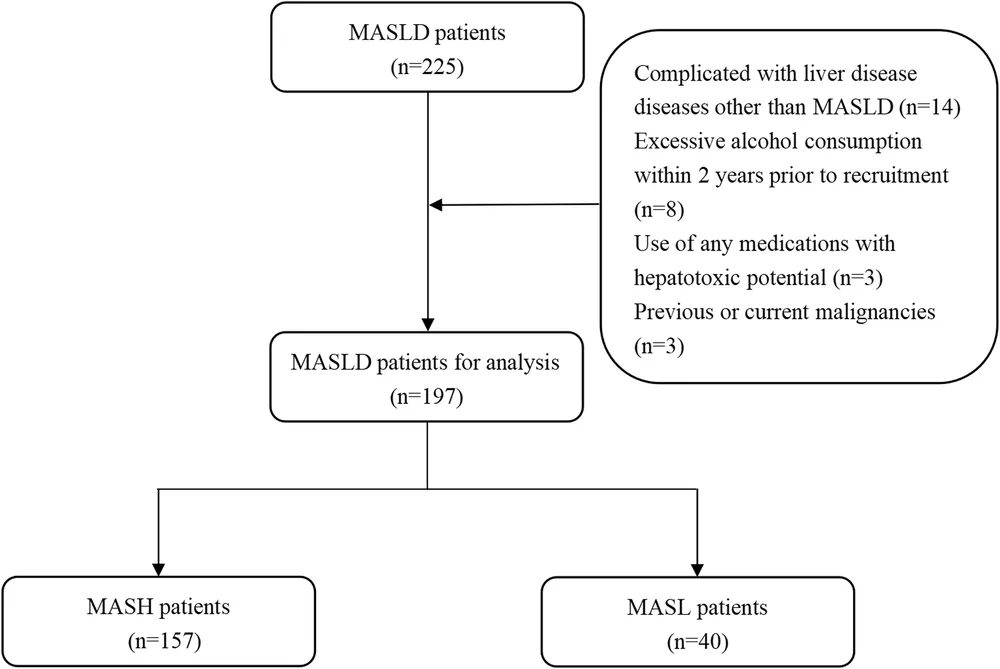
Patient flowchart

A total of 197 patients were included in the study, and the flowchart is shown in Fig. 2. According to the results of the histological evaluation, 157 patients were included in the MASH group, and 40 patients were included in the MASL group.

The clinical, serological, TE, and MRI data of the MASH group and MASL group are presented in Table 1. Significant intergroup differences were identified in multiple parameters, including sex, BMI, TG level, TC level, Cr level, eGFR, AST level, ALT level, GGT level, SATI, VATI, TATI, PDFF, LSM, and CAP (all *p* < 0.05).

**Table 1:** Comparison of clinical, serological, TE and MRI parameters between the MASH and MASL groups

Parameters	MASH (*n* = 157)	MASL (*n* = 40)	*p*
Age (years)	40.21 ± 11.26	41.55 ± 9.36	0.489
Sex (Male,%)	100 (64.69%)	33(82.50%)	**0.023**
BMI (kg/m^2^)	27.65 ± 3.79	25.70 ± 3.27	**0.005**
TG (mmol/L)	5.19 ± 1.02	4.76 ± 1.12	**0.021**
TC (mmol/L)	1.80 (1.34, 2.56)	1.76 ± 0.99	**0.031**
HDL (mmol/L)	1.07 (0.94, 1.23)	1.12 ± 0.28	0.575
LDL (mmol/L)	3.36 ± 0.94	3.16 ± 1.01	0.236
Glu (mmol/L)	5.18 (4.78, 5.74)	5.07 ± 0.70	0.094
HbA1c (%)	5.70 (5.40, 6.00)	5.75 ± 0.52	0.339
Cr (μmol/L)	70.82 ± 14.79	78.26 ± 14.41	**0.005**
eGFR (mL/min/1.73m^2^)	108.47 (99.03, 116.80)	101.94 ± 13.87	**0.014**
AST (U/L)	33.00 (22.00, 47.5.00)	23.97 ± 7.55	**< 0.001**
ALT (U/L)	52.00 (31.50, 91.50)	27.00 (19.00, 46.00)	**< 0.001**
TBIL (‌μmol/L)	11.70 (8.85, 14.55)	12.95 (8.60, 18.78)	0.120
Alb (g/L)	46.82 ± 3.36	47.15 (45.37, 49.53)	0.454
ALP (U/L)	72.00 (61.00, 81.00)	70.73 ± 18.52	0.402
GGT (U/L)	50.00 (33.50, 90.00)	36.50 (25.50, 49.75)	**0.002**
Waist circumference (cm)	911.99 ± 89.20	891.33 ± 73.40	0.199
SATI (cm^2^/m^2^)	55.71 (43.53, 74.11)	41.02 (33.60, 55.10)	**< 0.001**
VATI (cm^2^/m^2^)	62.95 (50.74, 74.19)	49.71 (42.38, 56.56)	**< 0.001**
TATI (cm^2^/m^2^)	123.59 (101.13, 143.34)	90.19 (80.97, 110.88)	**< 0.001**
PDFF (%)	20.36 ± 9.01	7.07 (5.18, 14.24)	**< 0.001**
LSM (kPa)	6.20 (5.00, 9.05)	5.30 (4.30, 6.68)	**0.005**
CAP (dB/m)	332.00 (290.00, 347.00)	277.03 ± 46.63	**< 0.001**
Steatosis Grade (*n*, %)			**< 0.001**
0	0	13	
1	42	26	
2	94	1	
3	21	0	
Lobular Inflammation (*n*, %)			**< 0.001**
0	0	3	
1	110	37	
2	47	1	
3	0	0	
Ballooning (*n*, %)			**< 0.001**
0	0	7	
1	51	33	
2	106	0	

Mann–Whitney *U*-test or independent samples *t*-test was used to compare the difference in continuous variables. The chi-square test was used to compare the differences in categorical variables

*MASH* metabolic dysfunction-associated steatohepatitis, *MASL* metabolic dysfunction–associated steatotic liver, *BMI* body mass index, *TG* triglyceride, *TC* total cholesterol, *HDL* high-density lipoprotein, *LDL* low-density lipoprotein, *Glu* fasting glucose, *HbA1c* hemoglobin A1c,* Cr* creatinine, *eGFR* estimated glomerular filtration rate, *AST* aspartate aminotransferase, *ALT* alanine aminotransferase, *TBIL* total bilirubin, Alb albumin, *ALP* alkaline phosphatase, *GGT* gamma-glutamyl transferase, *SATI* subcutaneous adipose tissue index, *VATI* visceral adipose tissue index, *TATI* total adipose tissue index, *PDFF* proton density fat fraction, *LSM* liver stiffness measurement, *CAP* controlled attenuation parameter

Bold values indicate *p* < 0.05.

### Model construction and robustness assessment for MASH identification

Univariate and multivariate logistic regression analyses revealed that PDFF (OR: 1.191, *p* < 0.001) and TATI (OR: 1.026, *p* = 0.001) were independent risk factors for MASH (Table 2). Based on the above logistic regression results, a combined MRI-based PDFF-Adipose Tissue (MR-PAT) model was constructed. The equation was as follows: Logit(P) = −3.917 + PDFF × 0.175 + TATI × 0.026.

**Table 2:** Univariable and multivariable logistic regression analyses for MASH

Variable	Univariable analysis		Multivariable analysis	
	OR	*p*	OR	*p*
Sex (Male,%)	0.372	**0.026**	-	-
BMI (kg/m^2^)	1.160	**0.006**	-	-
TG (mmol/L)	1.524	**0.023**	-	-
Cr (μmol/L)	0.966	**0.006**	-	-
eGFR (mL/min/1.73m^2^)	1.026	**0.038**	-	-
AST (U/L)	1.075	**< 0.001**	-	-
ALT (U/L)	1.035	**< 0.001**	-	-
SATI (cm^2^/m^2^)	1.031	**0.003**	-	-
VATI (cm^2^/m^2^)	1.042	**0.001**	-	-
TATI (cm^2^/m^2^)	1.031	**< 0.001**	1.026	**0.001**
PDFF (%)	1.196	**< 0.001**	1.191	**< 0.001**
LSM (kPa)	1.198	**0.016**	-	-
CAP (dB/m)	1.013	**0.001**	-	-

*BMI* body mass index, *TG* triglyceride, *Cr* creatinine, *eGFR* estimated glomerular filtration rate, *AST* aspartate aminotransferase, *ALT* alanine aminotransferase, *SATI* subcutaneous adipose tissue index, *VATI* visceral adipose tissue index, *TATI* total adipose tissue index, *PDFF* proton density fat fraction, *LSM* liver stiffness measurement, *CAP* controlled attenuation parameter

Bold values indicate *p* < 0.05.

**Fig. 3:**
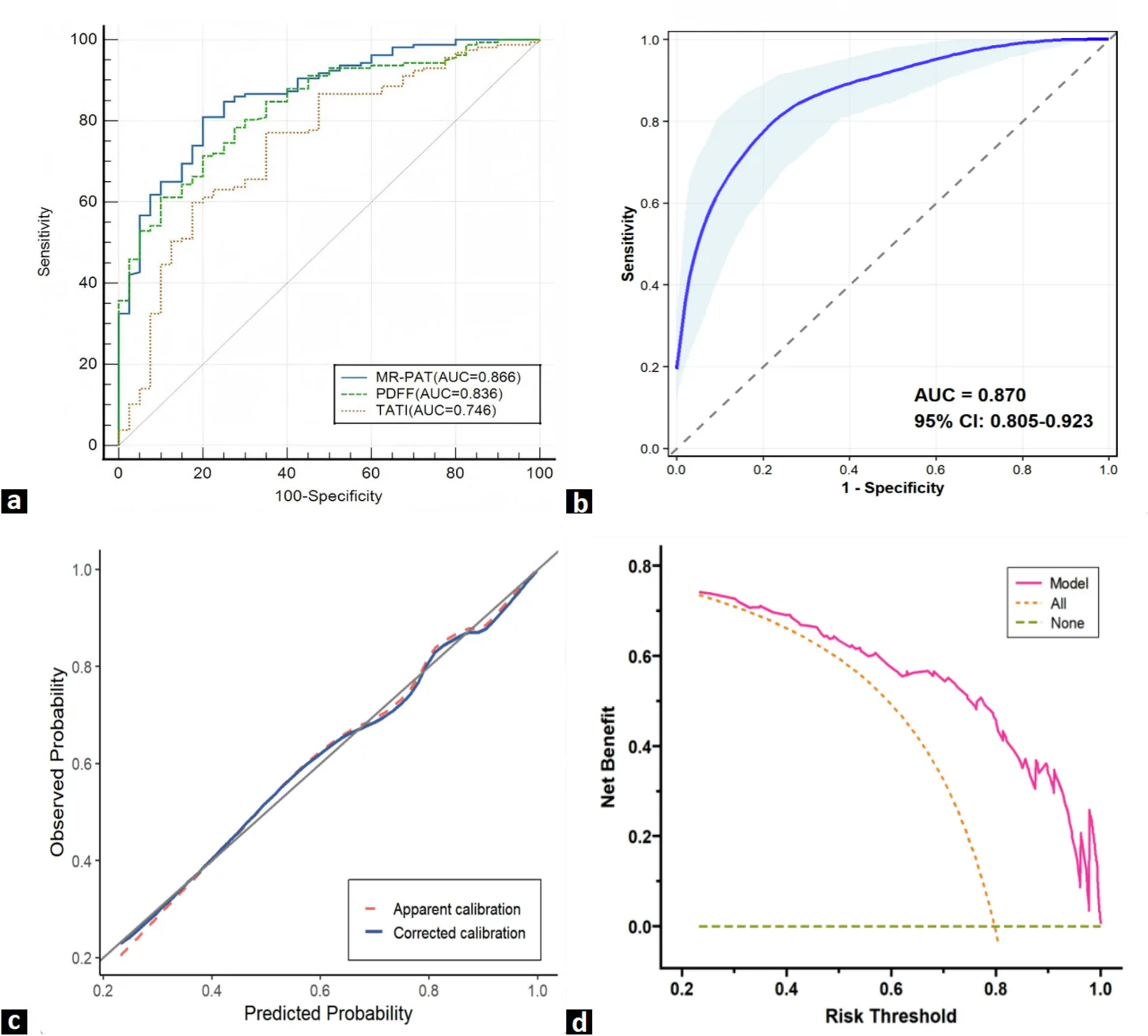
**a** ROC curves of the MRI-PAT model and its constituent variables. **b** Internal validation by bootstrap resampling; **c**, **d** Calibration curve and DCA curve of the model. PDFF, proton density fat fraction; TATI, total adipose tissue index

The AUCs of the MR-PAT model, PDFF, and TATI for identifying MASH were 0.866, 0.836, and 0.746, respectively (all *p* < 0.001) (Fig. 3a). Internal validation using bootstrap resampling of the MR-PAT model yielded an AUC of 0.870 (95% CI: 0.805–0.923, *p* < 0.001) (Fig. 3b). The calibration curve (Fig. 3c) demonstrated good agreement between the predicted and observed probabilities, which was supported by a nonsignificant Hosmer-Lemeshow test result (*p* = 0.672). DCA curves highlighted the substantial net clinical benefit of the combined model across a range of threshold probabilities (Fig. 3d).

We also established an additional model without PDFF. After PDFF was excluded from the candidate variable, CAP (OR: 1.007, *p* = 0.054), AST (OR: 1.057, *p* = 0.003) and TATI (OR: 1.024, *p* = 0.004) were selected (Supplemental Table S1). A combined CAP-AST-Adipose Tissue (CAAT) model was constructed. The AUCs of the CAAT model, CAP, and AST for identifying MASH were 0.816, 0.747, and 0.726, respectively (all *p* < 0.001). Bootstrap resampling yielded an AUC of 0.828 (95% CI: 0.757–0.891, *p* < 0.001), and the calibration curve and DCA curves are shown in Supplemental Fig. S1.

### Sensitivity analysis

**Fig. 4:**
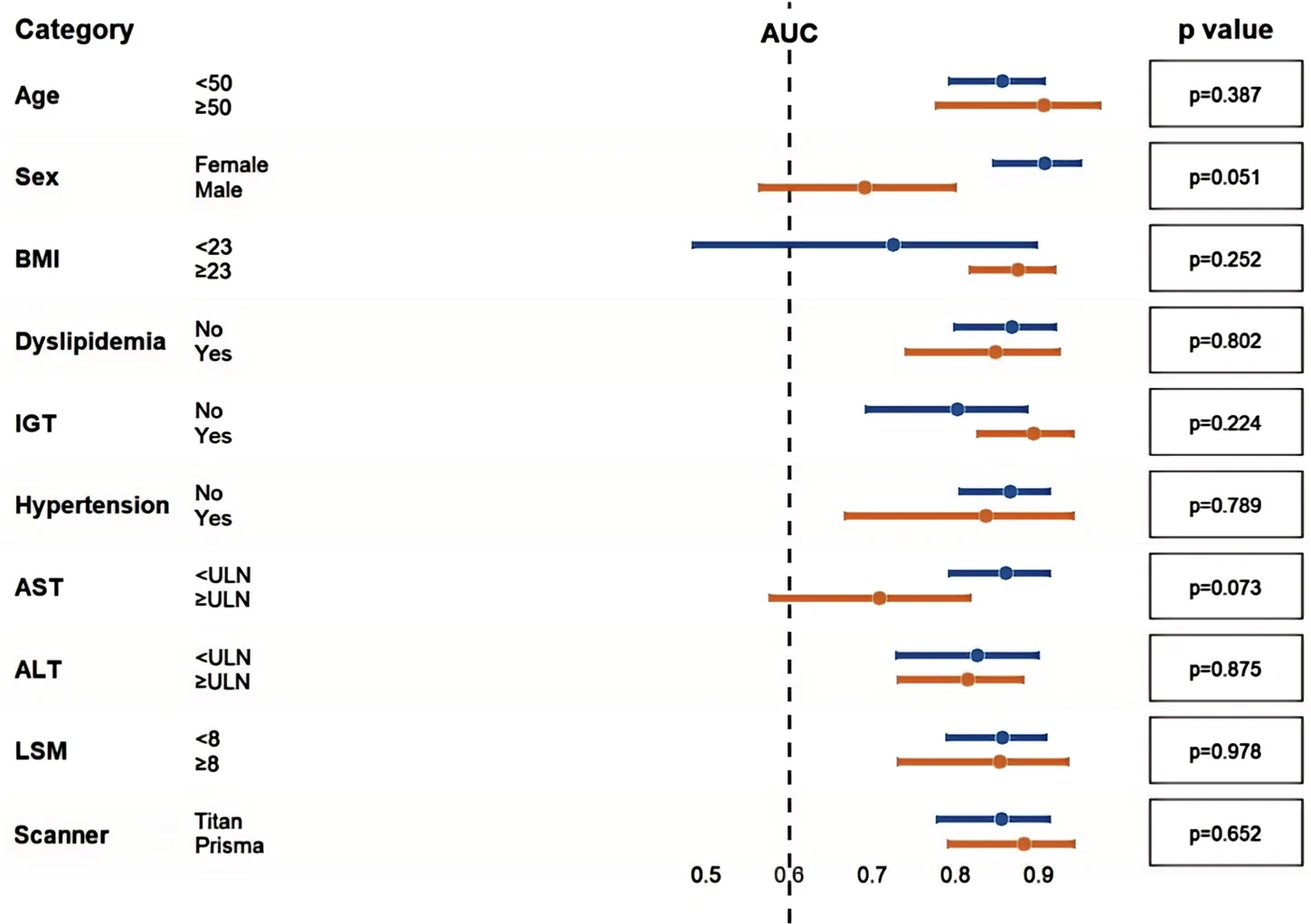
Sensitivity analysis of the AUC for MASH identification in different subgroups

Sensitivity analyses were performed to assess the diagnostic performance of the MR-PAT model in clinically relevant populations (Fig. 4). The results revealed no statistically significant difference in the model’s AUC across subgroups based on sex (male vs. female), age (< 50 vs. ≥ 50 years), BMI (< 23 vs. ≥ 23 kg/m²), atherogenic dyslipidaemia (yes vs. no), IGT (yes vs. no), hypertension (yes vs. no), ALT (< ULN vs. ≥ ULN), AST (< ULN vs. ≥ ULN), LSM (< 8 kPa vs. ≥ 8 kPa), or MRI scanner (Titan vs. Prisma).

### Comparison of the MR-PAT Model, PDFF, and NITs

The ROC curves for the MR-PAT model, PDFF, and NITs identifying MASH are summarized in Table 3. According to the DeLong test, the MR-PAT achieved a significantly greater AUC than FAST (*p* = 0.002), acNASH (*p* = 0.005), HSI (*p* < 0.001), FNI (*p* < 0.001), and APRI (*p* < 0.001). The AUC of the MR-PAT was greater than that of the PDFF, although the difference was not significant (*p* = 0.096).

**Table 3:** ROC curves for the MR-PAT, MRI-PDFF, and NITs for identifying MASH

	MR-PAT	PDFF	FSAT	acNASH	HSI	FNI	APRI
AUC	0.866	0.836	0.756	0.763	0.707	0.715	0.711
*p*-value	< 0.001	< 0.001	< 0.001	< 0.001	< 0.001	< 0.001	< 0.001
Rule out cut-off	0.57	8.44	0.06	2.24	34.20	0.19	0.17
Sensitivity (%)	90.4	90.4	91.7	90.4	90.4	91.7	91.7
Specificity (%)	55.0	55.0	17.5	20.0	42.5	17.5	12.5
NPV (%)	59.5	59.5	35.0	34.8	53.1	35.0	27.8
Gray-zone	60 (30.5%)	61 (31.0%)	96 (48.7%)	84 (42.6%)	120 (60.9%)	107 (54.3%)	96 (48.7%)
Rule in cut-off	0.89	17.9	0.37	4.53	46.09	0.66	0.35
Sensitivity (%)	61.8	60.5	49.0	55.4	26.1	42.0	50.3
Specificity (%)	92.5	90.0	90.0	92.5	90.0	90.0	90.0
PPV (%)	97.0	96.0	95.1	96.6	91.1	94.3	95.2

*AUC*, area under curve, *NPV* negative predictive value, *PPV* positive predictive value, *FAST* FibroScan-AST, acNASHAST to creatinine ratio index, *HSI* hepatic steatosis index, *FNI* fibrotic NASH index,* APRI* AST-to-platelet ratio index

In terms of the dual-cut-off strategy, compared with established NITs, MR-PAT resulted in a narrower gray zone, with a higher NPV at the rule-out cut-off and a higher PPV at the rule-in cut-off. Compared with the PDFF alone, the MR-PAT also showed a narrower gray zone and a higher PPV at the rule-in cut-off.

## Discussion

In this study, we developed a non-invasive MR-PAT model that combines MRI-based PDFF and TATI to identify MASH. Our results demonstrated that the MR-PAT model exhibited excellent diagnostic performance, with an AUC of 0.866, and significantly outperformed established NITs, including FAST, acNASH, HSI, FNI, and APRI.

Hepatic steatosis is not only the fundamental diagnostic criterion for MASLD but also serves as the pathophysiological basis of hepatocyte injury in steatohepatitis [3]. Excessive intrahepatic lipid accumulation induces lipotoxicity, triggering mitochondrial and endoplasmic reticulum stress and promoting reactive oxygen species (ROS) overproduction and lipid peroxidation [22, 23]. This lipotoxic stress directly damages hepatocyte membranes and organelles, driving hepatocyte ballooning and lobular inflammation [24]. This mechanistic framework explains why the hepatic fat content measured by the PDFF can serve as an independent predictor of MASH (AUC = 0.836). Beyond this, we further incorporated MRI-based adipose tissue to establish a combined model for MASH identification. Although the DeLong test revealed that the MR-PAT model did not yield a significant difference in the AUC compared with the PDFF alone (*p* = 0.096), the model yielded a narrower gray zone and a higher PPV at the rule-in cut-off. The implementation of a dual-cut-off strategy reduces diagnostic uncertainty by establishing clear rule-out and rule-in cut-offs, thereby facilitating the identification of patients who are potential candidates for therapeutic intervention or clinical trial enrollment. Moreover, adipose tissue quantification, which is available from standard MRI acquisition without extra sequences, adds diagnostic value to the PDFF in identifying MASH.

Multiparametric MRI biomarkers have demonstrated good diagnostic performance for identifying MASH. For instance, the combination of the PDFF and corrected T1 (cT1) has been reported to achieve AUCs ranging from 0.82–0.88 [25–27], which are comparable to those of our MR-PAT model. Nonetheless, cT1 measurement relies on an additional sequence and a proprietary algorithm, which may hinder its broader applicability. Marti-Aguado et al [16] recently developed a simple MR-MASH score that combines PDFF with waist circumference for noninvasively identifying patients with MASH, which shares a similar rationale with our study in leveraging adiposity-related parameters. Nevertheless, we did not calculate the MR-MASH score because the retrospective nature prevented waist circumference measurement at the same level. Instead, we obtained adiposity-related parameters at the more commonly used L3 level, but waist circumference did not differ significantly between the MASL and MASH groups. The reasons for the discrepancy in the relationship between waist circumference and MASH across the two cohorts remain to be investigated. However, waist circumference is a simple anthropometric measure that only indirectly reflects adiposity [28]. In contrast, TATI was defined as the aggregate of normalized visceral and subcutaneous adipose tissue areas, directly reflecting the adipose burden.

Excessive adipose tissue mass leads to adipocyte dysfunction, increased release of proinflammatory cytokines (e.g., TNF-α and IL-6), and reduced secretion of anti-inflammatory adipokines such as adiponectin [29–31]. These alterations promote hepatic insulin resistance, recruit inflammatory cells to the liver, and exacerbate hepatocellular injury [32, 33]. Interestingly, while both VATI and SATI were significant univariate predictors of MASH, TATI emerged as the independent factor in the final model. These findings suggest that compared with the distribution between the visceral and subcutaneous compartments, the cumulative burden of overall adipose tissue may be a more critical driver of steatohepatitis. As body composition analysis using MRI enables a direct evaluation of both the quantity and distribution of adipose tissue [34], this metric facilitates a more refined investigation of the impact of obesity on MASLD.

By excluding PDFF, we also constructed a CAAT model comprising TE-derived CAP, AST, and TATI. In this model, the CAP showed only a borderline association in the multivariable analysis (*p* = 0.054) rather than reaching conventional statistical significance. This finding does not necessarily invalidate its inclusion, given the relationship between CAP and hepatic steatosis and its clinical relevance. Notably, the two models share similar structural parameters, which further reinforces the value of incorporating both hepatic steatosis and adipose tissue in MASH screening. Thus, the difference in diagnostic performance between the models is likely attributable to the recognized superiority of the PDFF over the CAP in quantifying hepatic steatosis [35]. Accordingly, the MR-PAT model is recommended as the preferred non-invasive strategy when the PDFF is feasible, whereas the CAAT model may serve as a practical alternative under resource-limited conditions.

The diagnostic performance of our constructed model was compared with that of established NITs for identifying MASH, whereby the MR-PAT model yielded a significantly higher AUC. Among these NITs, FAST is of particular interest because it combines AST with TE-derived LSM and CAP—parameters that are widely used in MASLD risk stratification [7, 36, 37]. However, this comprehensive score showed only modest performance in our cohort. A probable explanation lies in FAST’s original design for identifying at-risk MASH, which anchors its algorithm to significant liver fibrosis in addition to MASH. This heterogeneity in histological endpoints may limit its ability to independently capture the histologic features of MASH. Our findings suggest that individualized screening strategies may be applicable according to the histological endpoint of interest. In clinical settings or trial designs focused on MASH identification, the MR-PAT model may offer the advantage of assessing MASH independently of liver fibrosis staging, potentially reducing misclassification among patients with early-stage MASH without significant fibrosis.

This study had several notable strengths. First, to our knowledge, this is the first study to demonstrate, using MRI, that hepatic steatosis and adipose tissue act synergistically to drive progression from isolated steatosis to steatohepatitis. Second, the MR-PAT model exhibited stable diagnostic performance across diverse subgroups, including patients examined on different MRI scanners, supporting its generalizability and potential for integration into clinical workflows. Third, the additional derivation of the CAAT model as an alternative for settings where the PDFF is unavailable further enhances the clinical utility of the adipose tissue quantification approach.

This study had several limitations. First, this retrospective study included a cohort from a tertiary care center, which may introduce selection bias, accounting for the low prevalence of MASL patients (20.3%) in our cohort. The generalizability of our findings to primary care screening populations warrants further investigation. However, given that patients with suspected MASL rarely undergo liver biopsy, the imbalance between the MASH and MASL groups in this study may reflect the real-world clinical workflow. Second, although bootstrap internal validation and varied comprehensive evaluations supported our model’s robustness, external validation is essential to confirm its generalizability in clinical practice. This is particularly critical given the relatively small sample size of our cohort. Therefore, the MR-PAT model requires rigorous evaluation in a large-scale, multicentre, external cohort validation before its clinical application. Third, while the MR-PAT model demonstrated robustness across different MRI manufacturers, it was confined to 3.0 T platforms. Nevertheless, the PDFF has been demonstrated to be highly reproducible between 1.5 T and 3.0 T scanners [38], and adipose tissue area measurements are inherently based on individual anatomy. This favors the cross-platform applicability of our model. Fourth, as a cross-sectional study, the longitudinal performance of our approach for predicting histological endpoints remains to be verified. Its long-term prognostic value for disease course will be among our priorities in future work.

In conclusion, the MR-PAT model, which combines MRI-based PDFF and TATI, leverages PDFF for accurate measurement of hepatic steatosis and TATI for direct quantification of adipose tissue burden, thereby providing a more precise reflection of the pathophysiological mechanisms driving steatohepatitis. It may serve as a non-invasive tool for risk stratification in patients with MASLD and holds promise for optimizing individualized management strategies.

## Supplementary information

ELECTRONIC SUPPLEMENTARY MATERIAL

## Data Availability

The data and material used and/or analyzed in this study are available from the corresponding authors on reasonable request.

## Abbreviations

Alb: Albumin
ALP: Alkaline phosphatase
ALT: Alanine aminotransferase
AST: Aspartate aminotransferase
BMI: Body mass index
CAP: Controlled attenuation parameter
Cr: Creatinine
eGFR: Estimated glomerular filtration rate
GGT: Gamma-glutamyl transferase
HbA1c: Hemoglobin A1c
HDL: High-density lipoprotein
LDL: Low-density lipoprotein
Glu: Fasting glucose
LSM: Liver stiffness measurement
MASH: Metabolic dysfunction-associated steatohepatitis
MASL: Metabolic dysfunction-associated steatotic liver
MASLD: Metabolic dysfunction-associated steatotic liver disease
PDFF: Proton density fat fraction
SATI: Subcutaneous adipose tissue index
TATI: Total adipose tissue index
TBIL: Total bilirubin
TG: Triglyceride
TC: Total cholesterol
VATI: Visceral adipose tissue index
