# Supplementary material for: MRI-based proton density fat fraction combined with adipose tissue for identifying metabolic dysfunction-associated steatohepatitis
Source: Insights Imaging. 2026 Oct 29;17:268. doi: 10.1186/s13244-026-02444-9 (PMC13719947; doi:10.1186/s13244-026-02444-9)
Supplement: Supplementary file 1 [file 13244_2026_2444_MOESM1_ESM.pdf]

MRI-based Proton Density Fat Fraction Combined with Aadipose Tissue for Identifying Metabolic Dysfunction-Associated Steatohepatitis

ELECTRONIC SUPPLEMENTARY MATERIAL

Insights Imaging (2026) Yang Y, Lai S, Li X et al.

Supplementary Method S1. CSE-MRI acquisition protocols across two MRI scanners

For the Siemens Prisma 3.0T scanner (Siemens Medical System): repetition time (TR)=9.0 ms; 6 echoes were collected in total, with first echo time (TE)=1.10 ms and interval ΔTE=1.23 ms; flip angle (FA)=4°; slice thickness=3.5 mm; field of view=38×31 cm; matrix=160×106; total acquisition time=15 seconds.

For the Titan 3.0T scanner (Toshiba Medical System): repetition time (TR)=5.1 ms; 2 echoes were acquired, with initial TE=1.1 ms and echo interval ΔTE=1.7 ms; flip angle (FA)=12°; slice thickness=8 mm; field of view=38×40 cm; matrix=256×192; scanning acquisition time=16 seconds. cm, and matrix 256×192. The entire acquisition procedure took 16 seconds.

Supplementary Table 1 Univariate and multivariate logistic regression analysis for MASH after excluding PDFF

Variable	Univariate analysis		Multivariate analysis	
	OR	P	OR	P
Sex (Male,%)	0.372	**0.026**	-	-
BMI (kg/m^2)	1.160	**0.006**	-	-
TG (mmol/L)	1.524	**0.023**	-	-
Cr (μmol/L)	0.966	**0.006**	-	-
eGFR（ml/min/1.73m^2）	1.026	**0.038**	-	-
AST (U/L)	1.075	＜**0.001**	1.057	**0.003**
ALT (U/L)	1.035	＜**0.001**	-	-
SATI (cm^2/m^2)	1.031	**0.003**	-	-
VATI (cm^2/m^2)	1.042	**0.001**	-	-
TATI (cm^2/m^2)	1.031	＜**0.001**	1.024	**0.004**
LSM (kPa)	1.198	**0.016**	-	-
CAP (dB/m)	1.013	**0.001**	1.007	0.054

BMI body mass index, TG triglyceride, Cr creatinine, eGFR estimated glomerular filtration rate, AST aspartate aminotransferase, ALT alanine aminotransferase, SATI：subcutaneous adipose tissue index, VATI visceral adipose tissue index, TATI total adipose tissue index, LSM liver stiffness measurement, CAP controlled attenuation parameter.

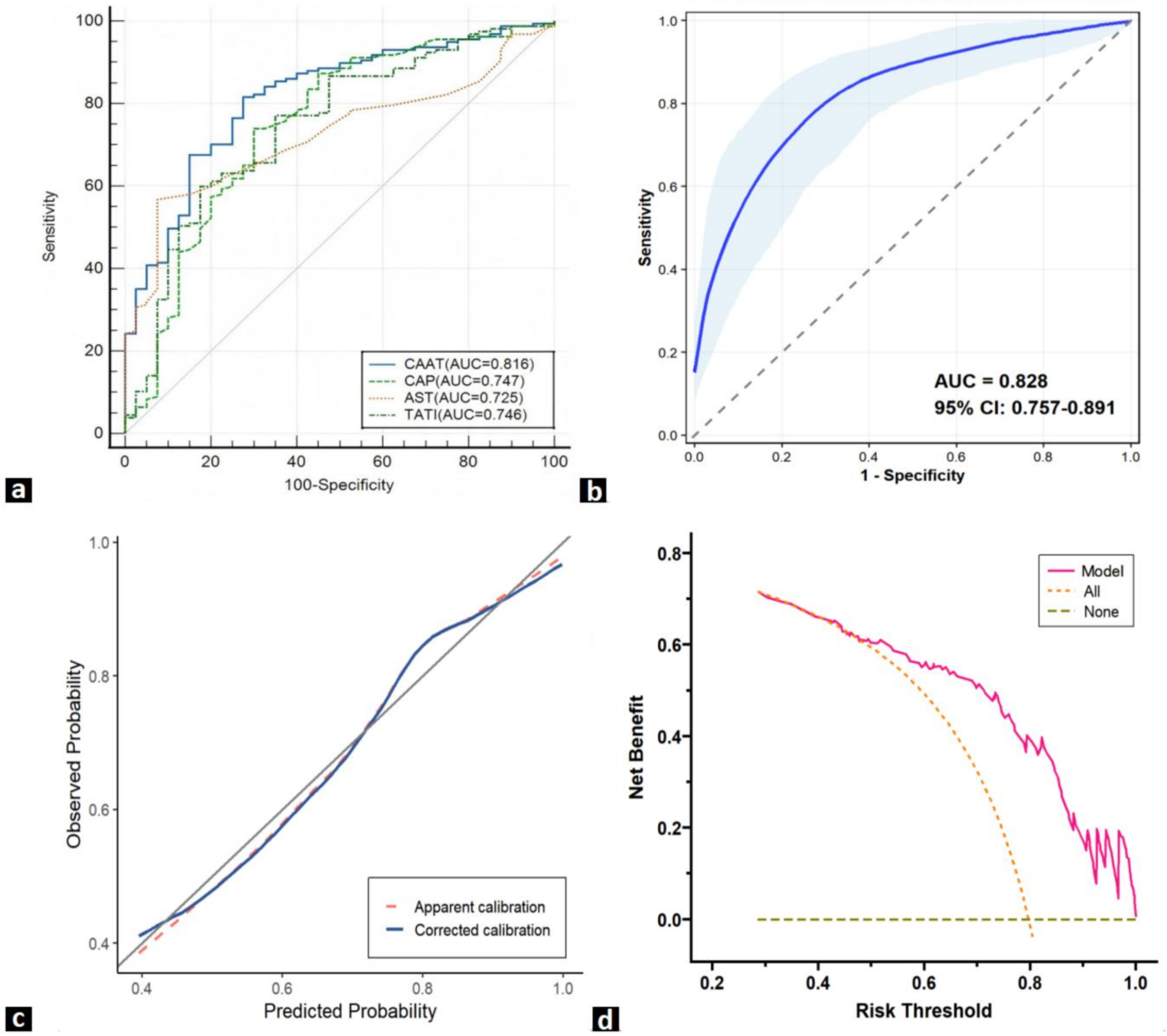

Supplementary Fig.1 (a) The ROC curves of the CAAT model and its constituent variables. (b) Internal validation by bootstrap resampling; (c, d) Calibration curve and DCA curve of the model. CAP controlled attenuation parameter, AST aspartate aminotransferase, TATI total adipose tissue index.
